# IMMUNOMETABOLIC CHECKPOINTS OF INFLAMMAGING

**DOI:** 10.1093/geroni/igad104.0458

**Published:** 2023-12-21

**Authors:** Vishwa Dixit

**Affiliations:** Yale University, New Haven, Connecticut, United States

## Abstract

Inflammation is a key hallmark and driver of age-related functional decline. Evidence that innate immune sensor NLRP3 inflammasome links aging to functional decline supports Metchnikoff’s original prediction that phagocytes or macrophages drive aging-associated degenerative diseases in an organism. Tissue resident macrophages are key to maintenance of homeostasis, a response that is compromised with age. It remains unclear whether negative energy balance in a host elicited by caloric restriction (CR) impacts mechanisms that control inflammation. The precise identity of factor(s) that link alterations in organismal metabolism to macrophage response are unclear. CR in rodents clearly elicits anti-inflammatory effects, but despite several years of research efforts, the endogenous drivers that couple energy balance to inflammatory cellular quiescence or activation is unknownThe RNA-sequencing analyses of adipose tissues from CALERIE-II study participants identified that CR in humans activates transcriptional programs implicated in mitochondrial metabolism, anti-inflammatory responses, and longevity. Our results show that macrophage derived protein PLA2G7, that is inhibited by CR in humans, reduces inflammaging, protects against thymic involution and protects against metabolic dysfunction. Furthermore, the CR in humans identifies unique regulators of inflammation including a CR-inhibited adipokine called SPARC that controls healthspan. Collectively, our studies establish that CR is relevant to human physiology and immunobiology and offers an important platform to harness immunometabolic checkpoints of inflammation and longevity.

